# Diverse data supports the transition of filamentous fungal model organisms into the post-genomics era

**DOI:** 10.1080/21501203.2017.1281849

**Published:** 2017-02-17

**Authors:** Kevin McCluskey, Scott E. Baker

**Affiliations:** a Department of Plant Pathology, Kansas State University, Manhattan, KS, USA; b Environmental Molecular Science Laboratory, Pacific Northwest National Laboratory, Richland, WA, USA

**Keywords:** Filamentous fungi, gene-for-gene hypothesis, bioprocessing, biotechnology, parasexual genetics, genetic maps, molecular markers, historical genetics

## Abstract

Filamentous fungi have been important as model organisms since the beginning of modern biological inquiry and have benefitted from open data since the earliest genetic maps were shared. From early origins in simple Mendelian genetics of mating types, parasexual genetics of colony colour, and the foundational demonstration of the segregation of a nutritional requirement, the contribution of research systems utilising filamentous fungi has spanned the biochemical genetics era, through the molecular genetics era, and now are at the very foundation of diverse omics approaches to research and development. Fungal model organisms have come from most major taxonomic groups although Ascomycete filamentous fungi have seen the most major sustained effort. In addition to the published material about filamentous fungi, shared molecular tools have found application in every area of fungal biology. Similarly, shared data has contributed to the success of model systems. The scale of data supporting research with filamentous fungi has grown by 10 to 12 orders of magnitude. From genetic to molecular maps, expression databases, and finally genome resources, the open and collaborative nature of the research communities has assured that the rising tide of data has lifted all of the research systems together.

## Introduction

Because fungi were attractive models for studying genetic phenomena, they were extensively utilised during the early classical genetic era. Different systems were utilised, with differing applications, and some of these have persisted into the modern era. Among the earliest systems, water moulds such as *Blastocladiella* (Blackwell 1939) and *Allomyces* (Emerson & Fox 1940) were used to understand mating and pigment production. While auxotrophic mutants were generated in *Allomyces*, genetics were complicated by the alternation of generations and tendency to generate autotetraploids. In keeping with progress in diverse research areas, fungal chromosome were targeted for analysis early in the classical genetics era (Subramaniam & Ranganathan 1946) and revealed a similarity between bacterial and fungal chromosomes (Lindegren 1948) ultimately leading to characterisation of fungal karyotypes in *Sordaria* (Carr & Olive 1958) and *Ceratocystis* (Aist 1969). Fungal model organisms soon came to the attention of classical cytologists and led to the observation of *Neurospora* (McClintock 1945) and *Aspergillus* (Elliott 1960) chromosomes. At this time, there were few data points for each organism: genus, species, host, morphology, colour, culture requirements, mating type, number of chromosomes, and applied use.

Yeast was utilised from an early date, with studies of mating and life cycle (Lindegren 1945) leading to establishment of mating systems (Lindegren & Lindegren 1943), as well as early elucidation of growth requirements (Williams et al. 1940). Similarly, research with basidiomycetes led to the development of auxotrophic mutant analysis (Perkins 1949a) in the corn smut pathogen *Ustilago maydis* and mating, or incompatibility factors, in *Schizophyllum commune* (Papazian 1950). These systems were utilised to elucidate self-recognition systems and have spanned the classical genetics and molecular genetics eras (Banuett 2015). However, the emergence of filamentous fungi as model organisms built upon the special characteristics such as ease of manipulation, mutation, and genetic mapping via sexual and parasexual genetics in *Neurospora crassa* (Beadle & Tatum 1941) and *Aspergillus nidulans* (Pontecorvo et al. 1953a), respectively. The ability to utilise ordered asci to study second division segregation in *Neurospora* was a major advantage in understanding gene order and position of centromeres and allowed rapid identification of chromosome aberration mutants (Perkins 1974).

Sharing of data was a hallmark of these research communities and led to rapid advances in many areas. Published and curated genetic maps (Pontecorvo et al. 1953b; Barratt et al. 1954b) allowed coordinated approaches that were facilitated by open meetings, such as the *Neurospora* Information Conference which was formalised in 1961 (1962) and continues today as the Genetics Society of America Fungal Genetics Conference at Asilomar (Momany et al. 2015). Both the *Neurospora* and *Aspergillus* communities had shared newsletters and these have merged and matured to form the peer-reviewed journal, *Fungal Genetics Reports*. In the pre-internet era, these information portals were the main conduit for free and open exchange of information. Formally a supplement to the Neurospora Newsletter, continuing as this became the Fungal Genetics Newsletter, and finally giving way to the electronic version, the Fungal Genetics Stock Center (FGSC) catalogue was published every other year and included publication of genetic maps. Even the organisation of the FGSC catalogue reflected the genetic location of markers in the genetic map with the left-most marker (*ro-10*) on Linkage Group I being the first entry in the multiply marked mutant section and the farthest-right marker on Linkage Group VII (*nt)* being the last (Mccluskey & Plamann 2006). This free exchange of information allowed these key model organisms to flourish and had impact on the growth of the research communities and how they translated to applications in agriculture, food and fibre processing, and biotechnology. Building upon the success of work with model organisms, researchers have applied the approaches validated in these more simple organisms to plant and human pathogenic fungi with great success. Progress in these systems, more accurately called research systems to reflect their practical importance in agriculture and health, has been rapid and in many cases bypassed the laborious phenotype based or even molecular marker-based genetic mapping to go directly to genome informed approaches (Ma et al. 2010; Couturier et al. 2012; Schmoll et al. 2016).

### Neurospora crassa

**Figure 1.**
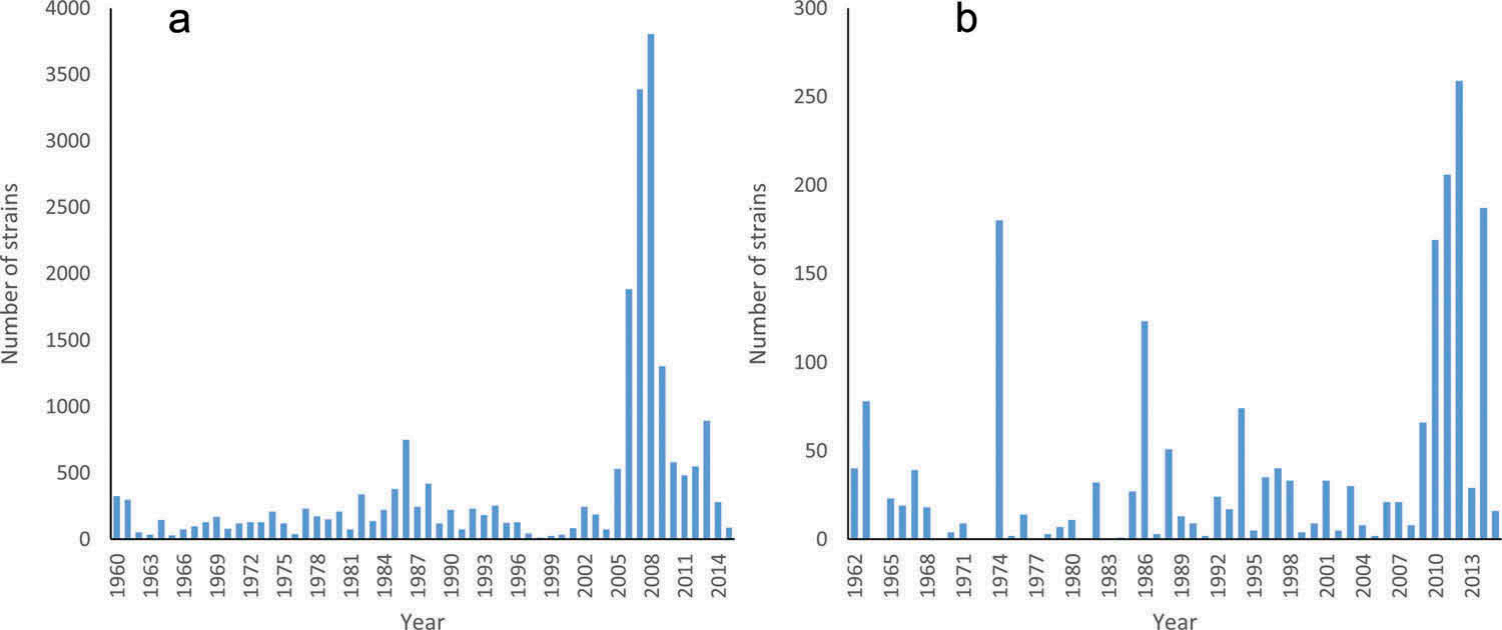
Growth of holdings at the FGSC as annual deposits: (a) *Neurospora*; (b) *Aspergillus*.

From its origins as a well-behaved organism in which to do genetic crosses that demonstrated the difference between heterothallic and homothallic species (Shear & Dodge 1927), *N. crassa* soon became a model for a diversity of biological questions. Among these, the seminal demonstration of the one-gene, one-enzyme hypothesis (Beadle & Tatum 1941) led the way to the construction of a densely populated genetic map (Perkins et al. 1962b; Nelson & Perkins 2000) including over 1000 phenotypic markers and more molecular, chromosomal, and cytogentic markers (Perkins et al. 2001). As a leading model for the development of technologies important to industrial biology (Kato & Stuart 1998), plant pathology (Pandey et al. 2004), and even human physiology (Dunlap 1999), Neurospora was first among filamentous fungi to have a fully sequenced and annotated genome (Galagan et al. 2003). Going from 1500 genetic markers and the associated data to a fully annotated genome sequence represented a 10-fold increase in data about genes, and six orders of magnitude increase in the data describing the reference genome strain. The use of these strains with a common genetic background leveraged the impact of this genome data and helped keep *Neurospora* as a key model system for many areas of biological research. Organised sharing of strains through the FGSC supported the use of the shared genetic background (McCluskey 2003). The growth of holdings in the *Neurospora* collection at the FGSC has been impacted by organised deposits, beginning with the accession of classical mutants in the 1960s and then strongly by the deposit of targeted gene deletion mutants through collaboration with the *Neurospora* functional genomics project (Colot et al. 2006) (Figure 1). Overall, 532 individuals have deposited strains into the FGSC collection.

*Neurospora* has long been used as a genetic model (Davis & Perkins 2002) and this may be its most significant long-term impact (Roche et al. 2014a). Many early studies focused on mutation and mutability (De Serres & Kolmark 1958; De Serres & Brockman 1999) and, for example, the reference genome strain is called “Oak Ridge” reflecting its origins as a model organism for the study of low-dose radiation at the Atomic Energy Commission (a predecessor to the current US Department of Energy) Oak Ridge National Laboratory (Kafer 1982). As a model for cell biology and physiology, Neurospora has allowed significant impact in understanding of genome defence through vegetative gene silencing (Fulci & Macino 2007), meiotic gene silencing (Shiu et al. 2001; Shiu & Metzenberg 2002), mutation of repeated DNA during meiosis (Selker & Garrett 1988; Cambareri et al. 1991), and overall epigenetic gene regulation (Cambareri et al. 1996; Selker et al. 2003; Aramayo & Selker 2013). As a model for molecular physiology, it has been one of the leading models for analysis of the endogenous circadian rhythm (Baker et al. 2012).

While it was long thought that *Neurospora* was not an important economic platform, the numerous patents for protein expression (Kato & Stuart 1998), for the production of heterologous dimeric proteins (Stuart 1997), and even vaccine production (Allgaier et al. 2009) demonstrate the utility of this well-studied system. More recently Neurospora has been investigated as a model for ​biomass decomposition (Znameroski et al. 2012), biofuel production (Roche et al. 2014b), and even for production of gold and other metal nanoparticles (Castro-Longoria et al. 2011). Over 1000 patents in the US Patent and Trademark Office (USPTO) ‘Global Patent Search Network’ (http://gpsn.uspto.gov/) list *Neurospora*. Increasing rapidly after publication of the genome in 2005, in 2007 there were 126 patents citing Neurospora (Table 1). The availability of diverse tools, the rapid, unrestricted growth, and the general accepted safety profile (Perkins & Davis 2000) make *Neurospora* an attractive system to study these practical and important topics.

**Table 1.** Numbers of patent citing model filamentous fungi.

Year	*Neurospora*	*Aspergillus*	*Trichoderma*
1986	3	19	4
1987	1	22	6
1988	1	24	11
1989	2	34	12
1990	2	39	18
1991	1	40	10
1992	0	43	18
1993	1	53	15
1994	2	82	18
1995	2	69	16
1996	4	121	38
1997	11	145	42
1998	14	189	50
1999	26	241	102
2000	30	281	90
2001	42	407	115
2002	37	477	145
2003	40	499	126
2004	49	620	188
2005	80	831	224
2006	94	919	260
2007	126	1,013	331
2008	121	1,044	311
2009	80	573	174
2010	109	1,096	382
2011	86	855	296
2012	2	196	81
2013	38	1,311	552
2014	6	374	155

Source: US patent and trademark office global patent search network, http://gpsn.uspto.gov/).

*N. crassa* was among the first fungi in which molecular transformation was accomplished, first by demonstration of supplement independence in inositol requiring mutants (Mishra & Tatum 1973), and soon by explicit complementation of auxotrophic mutations including the *qa-2* mutant in an *arom-9* background (Case et al. 1979), and glutamate dehydrogenase (*am*) (Grant et al. 1984; Kinsey & Rambosek 1984). Transformation protocols have varied over time, and reflecting the availability of key reagents, such as cell-wall degrading enzymes, and include protoplast transformation (Radford et al. 1981; Buxton & Radford 1984), lithium acetate treatment of germinated conidia (Dhawale et al. 1984), and most recently electroporation (Chakraborty et al. 1991). While most transforming vectors for Neurospora integrated into the nuclear genome, a system for transformation based on autonomous replication in host mitochondria was developed (Stohl & Lambowitz 1983). *Neurospora* researchers were able to generate targeted mutation of genes by use of the endogenous Repeat Induced Point Mutation process (Selker & Garrett 1988; Cambareri et al. 1991). Other targeting systems, including targeting to the *his-3* locus (Aramayo & Metzenberg 1996), overcame some of the problems with multiple integrations found with earlier systems. For example, the use of the microtubule disrupting antibiotic Benomyl for selection of strains carrying a mutant version of the beta-tubulin gene led to multiple integrations as well as genome instability (Orbach et al. 1986). Beyond this, recovery of strains carrying chromosome rearrangements in as many as 10% of transformants demonstrated the mutagenicity of transformation (Perkins et al. 1993) even for selectable markers not associated with microtubule function. This was a limiting factor for many studies and several investigators explored the use of cells deficient for non-homologous end-joining to take advantage of high locus-specific integration by the homologous recombination pathway. Inoue and colleagues were the first to demonstrate that nearly 100% targeting of transforming DNA could be accomplished in the transformation of *N. crassa* strains with defective copies of the Ku70 or Ku80 genes (*mus-51* and *mus-52*) (Ninomiya et al. 2004) building upon many years of study of genes for mutation repair (Inoue et al. 1981).

Being a highly collaborative community, curated genetic maps were shared and published in the *Neurospora* Newsletter and the catalogue of the FGSC (Mccluskey & Plamann 2006). The annual Neurospora bibliography (Strickland) provided another unparalleled data resource prior to the development of robust and open bibliographic databases such as Google Scholar. Shared data and molecular resources such as an restriction fragment length polymorphism​ mapping population and data (Nelson & Perkins 2000), gene (Nelson et al. 1997), and genome libraries (Kelkar et al. 2001) facilitated the development of one of the most densely populated genetic maps for all organisms (Barratt et al. 1954a; Perkins et al. 1969). Over many years, the genetic map along with characteristics of the mutants and mapping data have been published as a gene compendium (Perkins et al. 1982, 2001). This compendium is available electronically at Leeds University and includes a database of gene names for genes discovered only by genome annotation and comparison with other organisms (Radford 2013).

After the publication of the *Neurospora* genome (Galagan et al. 2003), there was rapid development of genome-enabled technologies including a robust single-nucleotide-polymorphism-based map (Lambreghts et al. 2009), DNA oligonucleotide micro-arrays (Kasuga et al. 2005) supported by shared RNA expression data, and now the availability of targeted gene-deletion mutants at almost every locus (Colot et al. 2006) which effectively provides a drug-resistance marker suitable for genetic mapping (Hammond et al. 2012) across the genome. Currently *Neurospora* has nearly 2000 genome and transcriptome projects published in the National Center for Biotechnology Information Short Read Archive and over 600 strains in the FGSC collection have been subject to transcriptome or genome sequence analysis including wild-type (Ellison et al. 2011a) and classical mutant strains (McCluskey et al. 2011). The impact of shared resources has reinforced *Neurospora* as a model for plant biomass deconstruction (Znameroski et al. 2012), plant–microbe interactions (Kuo et al. 2014), and a myriad of areas of cell biology and physiology (Roche et al. 2014a). Most genome resources for *Neurospora* were originally maintained at the Broad Institute, although as of 2016 these are only available as an historical archive (http://archive.broadinstitute.org/ftp/pub/annotation/fungi/neurospora_crassa/). Subsequent to the availability of genome information via the Broad Institute, the US Department of Energy Joint Genome Institute (JGI) has developed and maintains extensive genome resources for Neurospora and other fungi as part of their Mycocosm resource (http://genome.jgi.doe.gov/programs/fungi/index.jsf) (Grigoriev et al. 2013). For example, they host the genome sequence of the only *N. crassa* strain with the mating type “a,” important because the mating loci are non-homologous (Baker et al. 2015). Additional *Neurospora* species genome sequences are available through the JGI including *N. tetrasperma* (Ellison et al. 2011b) and *N. discreta* (http://genome.jgi.doe.gov/Neudi1/Neudi1.home.html).

## *Aspergillus* species

### Aspergillus nidulans

Originally isolated as a stable system to do parasexual genetics (Pontecorvo 1946; Pontecorvo et al. 1953a), the demonstration of sexual genetics (Hemmons et al. 1952) made mapping more straight forward, and allowed direct comparison to genetic maps made in other organisms. Having been originally described as *Sterigmatocystis* in 1883 (Eidam), *A. nidulans* was revised by Thom and Raper (1939) based upon primarily morphological characteristics of the conidia, sterigmata, and perithecia. Advances in the *Aspergillus* research community grew along the complimentary tracks of industrial utilisation for bioprocessing (Birkinshaw et al. 1931), clinical characterisation of pathogenic and immunologically reactive isolates (Wright 1927; Puestow 1929; Brown 1932), and through the production of mutants (Hockenhull 1948), in morphological (Raper et al. 1945), biochemical (Pontecorvo 1949), and physiological traits (Roper & Käfer 1957; Lanier et al. 1968). Among the latter, cytoplasmic mutations were characterised in *A. nidulans* by ultraviolet light irradiation (Arlett 1957). Ultimately, there were hundreds of physiological, biochemical, and morphological mutants available through collaboration between the Glasgow (Clutterbuck 1969) collection and the FGSC, and research with this system proved invaluable to subsequent systems for industrial, medical, and agricultural applications.

*A. nidulans* has always been primarily a research organism with little direct economic impact (May & Adams 1997) although its ease of culture (Thom & Church 1921), maintenance (Greene & Fred 1934), and manipulation (Pritchard 1955; Tilburn et al. 1983) firmly established *A. nidulans* as the leading model for understanding and exploitation of fungi in the Eurotiales. Because of this leading role, diverse strains and related tools for manipulation of *Aspergillus* are available. The merging of the FGSC and Glasgow collections led to the FGSC as the central resource for *Aspergillus* research. Beginning with the deposit of 41 mutant strains by E. Kafer in 1962, the *Aspergillus* collection at the FGSC had grown to include over 2000 strains in 2015 (Figure 1(b)).

Additional tools for understanding *A. nidulans* have included shared genome (Brody et al. 1991) and cDNA (Kupfer 1999) libraries, cloning vectors and transformation vectors (Oakley et al. 1987). Conveniently, most laboratories work with strains in the same genetic lineage, and the reference strain, FGSC A4, was used for mutant construction and molecular analysis and was the first *A. nidulans* strain to have its genome sequenced. This genome assembly took advantage of genome libraries from the FGSC collection to order the contigs (Galagan et al. 2005) and the genome coordinates of clones in these libraries was published at the genome website allowing rapid access to specific regions of the genome. The availability of strains deficient in non-homologous recombination has allowed targeted transformation with great fidelity, and as part of a collaborative programme, gene deletion cassettes for every gene in the annotated online genome are available through the FGSC (De Souza et al. 2014). Deposit of strains generated with these molecular tools has been slow, but the number of publicly available *Aspergillus* strains in the FGSC collection has more than doubled since 2000 (Figure 1(b)).

Because stable introduction of DNA sequences was of paramount importance, early efforts to enable DNA-based transformation relied on stably integrating DNA vectors. Early vectors carried wild-type copies of biosynthetic genes, such as *ArgB* (John & Peberdy 1984), although there were many abortive transformants and the ultimate yield of stable transformants was on the order of 50 per microgram of transforming plasmid DNA. Among these, some were unstable and apparently contained multiple integrations. Complementation of *argB* was employed in numerous *Aspergillus* species. Selection of transformants carrying a wild-type copy of the acetamidase structural gene in strains with a deletion of the native acetamidase gene allowed recovery of up to 25 transformants per microgram of transforming DNA (Tilburn et al. 1983). Additional auxotrophic markers, such as *trpC* (Yelton et al. 1984), and *pyrG* (Ballance et al. 1983) expanded the impact of Aspergillus as a research system. ​Most modern transformation systems use complementation of *pyrG89*. Transformation of wild-type strains being desirable, a drug-resistance marker was sought and ultimately transformation with selection for the presence of the *Escherichia coli* hygromycin resistance gene was developed (Punt et al. 1987), although this marker is more widely used in other systems because of high innate resistance in Aspergilli. Autonomously replicating vectors for *Aspergillus* allowed recovery of up to 250 transformants per experiment (Gems et al. 1991) and formed the basis of gene libraries (and vectors) deposited into the FGSC collection and with diverse characteristics (Osherov & May 2000). The *A*. nidulans genome was publicly sequenced in 2005 (Galagan et al. 2005) and combined with diverse tools for *A. nidulans* and other Aspergilli insures that this will be an important system for years to come (Bennett 2009). Further supporting the availability of diverse mutants, a gene deletion cassette library for *A. nidulans* was generated and made publicly available through the FGSC (De Souza et al. 2013).

Data resources for work with *A. nidulans* began with strains from the Glasgow collection serving as a foundation and rapidly grew to include a densely populated genetic map. Originally based on parasexual genetics (Käfer 1958; Pontecorvo & Käfer 1958) and later incorporating meiotic mapping data (Dorn 1967), the genetic map made the assembly of the sequenced genome robust and authoritative (Galagan et al. 2005). Other data resources for *A. nidulans* include a curated gene compendium (http://www.fgsc.net/Aspergillus/gene_list/index.html) and annual bibliography (http://www.fgsc.net/scripts/biblioNEW.asp) as well as collaborative genome tools, including a central data repository (Mabey et al. 2004) and also the *Aspergillus* Genome Database (Arnaud et al. 2012). With a general move away from organism-specific database resources, the *A. nidulans* community has migrated to the fungiDB integrated genomics database for fungi (Stajich et al. 2012).

### Aspergillus oryzae

While research on *A. nidulans* and *N. crassa* were being conducted in Scotland and the USA, research into the biology and genetics of *A. oryzae* was well underway in Japan. Among the earliest studies with filamentous fungi, research into the breakdown of starch by koji fungus was described as early as 1889 when the organism *A. oryzae* was described in a patent (Takamine 1889) that documented increases in the final alcohol content based upon increases in sugar content of the starch feedstock. *A. oryzae* was formally named in 1921 (Thom & Church 1921) and was extensively studied both with regard to its ability to saccharify starchy substrates and also for its production of kojic acid (Yabuta 1924).

The use of *A. oryzae* in research was primarily in regard to its industrial applications, such as the production of vitamins (Takahashi & Lim 1929), phytase (Kawahara 1929), and for its use in standardising biochemical activities especially amylases (Horton 1921). While investigated for its amenability for parasexual genetic analysis in the 1940s (Pontecorvo 1946), it was not until the 1950s that *A. oryzae* was extensively characterised genetically (Ishitani et al. 1956). Soon thereafter, studies of morphological mutants (Ishitani & Sakaguchi 1955) and biochemical pathways in *A. oryzae* were published, the latter with emphasis on unique metabolites in the organism (Ikeda 1958). Studies of auxotrophic mutants of *A. oryzae* were described in 1958 and emphasised vitamin and pigment mutants (Fukami 1958). The new discoveries of genetic and para-genetic tools for *A. oryzae* were rapidly applied to strain improvement (Ikeda et al. 1957). Because of the highly applied aspect of research with *A. oryzae*, genetic maps were not readily available and it was not until the publication of an electrophoretic karyotype in the early 1990s that the genome of this organism was characterised (Kitamoto et al. 1994). This work showed the location of key genes including various amylases, nitrate reductase, tubulin, and ribonuclease T1 on various chromosomes and highlighted the low repeat content in the genome. A whole genome sequence for *A. oryzae* was published in 2005 (Machida et al. 2005).

Always used in beverage, chemical, and enzyme production, *A. oryzae* was rapidly utilised as a host for foreign gene expression (Gomi et al. 1987) including the early expression of calf chymosin (Tsuchiya et al. 1994). *A. oryzae* strains have been used for expression of diverse foreign genes including manganese peroxidase (Stewart et al. 1996), fatty acid desaturases (Sakuradani et al. 1999), lipases(Høegh et al. 1995), and more recently strains deficient in proteases have been utilised for expression of proteins destined for secretion and subsequent purification (Yoon et al. 2011).

Early transformation of *A. oryzae* was accomplished using the *argB* gene (Gomi et al. 1987), although like for *A. nidulans* multiple integrations were common. While the yield was fewer than one transformant per microgram of plasmid DNA, these transformants were stable for multiple transfers without selection. At the same time, transformation was accomplished by complementation of *pyrG* (Mattern et al. 1987), met (Iimura et al. 1987), and later *niaD* (Unkles et al. 1989). Similarly, dominant selectable markers based upon carboxin resistance (Shima et al. 2009) and recyclable markers (Maruyama & Kitamoto 2008) as well as strains for targeted transformation (Takahashi et al. 2006) based upon the production of non-homologous-integration-deficient strains has facilitated use of *A. oryzae*. Most recently, the use of the genome editing techniques has been expanded to this fungus (Katayama et al. 2016) promising a valuable future. As much as *A. oryzae* is an important applied and model system, there are not so many public resources. Collections hold diverse strains, although they are not numerically abundant. The FGSC holds only three strains, the Centraalbureau voor Schimmelcultures (CBS) collection 23 and the US Department of Agriculture, Agricultural Research Service “NRRL” ​ collection 19. The Straininfo.net resource lists 352 *A. oryzae* strains in culture collections worldwide although these numbers are complicated by somewhat fluid taxonomic designations. Some strains are listed as subspecies of *A. flavus*, for example.

### Aspergillus niger

Because *A. niger* was ubiquitous on grain, fruit, and vegetable products, it was brought into cultivation very early and was among the first fungi to have defined growth requirements (Raulin 1869). Soon recognised as producing invertase (Bay 1894), inulinase (Dean 1903), and protease (Wilson 1930), *A. niger* was also implicated as contributing to animal and human infection (Boyce & Surveyor 1894; Hatch & Row 1900), although the taxonomy of the time does not differentiate among black aspergilli. *A. niger* was characterised for its ability to be cultured in diverse formats (Le Mense et al. 1947), and the collection of industrial strains at the US Department of Agriculture Northern Region Research Laboratory became well recognised for the valuable resources held there (Raper & Alexander 1945). Among these, strain NRRL 3 is one of the most well utilised and studied strains. It has given rise to a myriad of offspring and is held in multiple collections (see, for example, the strain table (Andersen et al. 2011)).

Among the most widely utilised fungi in industrial and biological processes, *A. niger* has been called a cell factory (Pel et al. 2007) to reflect its widespread adoption and impact. Because of its importance for both enzyme and organic acid production, multiple strains of *A. niger* have sequenced and analysed (Pel et al. 2007). The reference sequence data from these strains has been critical for development of ‘fast forward’ genetic approaches for association of genes with mutant phenotypes (Niu et al. 2016a, 2016b). All of this has built upon early sharing of research tools. For example, the deposit of a set of genetically characterised mutant strains in the FGSC collection by C.J. Bos in 1986, by K. Swart in 1994, and by E. Kafer in 1996, led to the availability of a well-characterised lineage of mutant strains as a valuable reference for industrial development in *A. niger*. With holdings of 145 mutant and seven wild-type strains, *A. niger* is among a small group of model organisms with a robust and public genetic foundation. Other collections hold significant numbers of *A. niger* strains, although often of industrial application rather than of genetic emphasis. The NRRL collection lists 37 *A. niger* strains and the CBS collection, 81 strains. Many of these strains are held by multiple collections and the Straininfo.net resource lists over 900 *A. niger* strains, again with many having been deposited under various names in collections around the world.

As for *A. nidulans* and *A. oryzae*, molecular transformation followed a natural path of complementation of auxotrophic mutants (Buxton et al. 1985) and, ultimately, selection of Hygromycin resistant colonies at rates of 5–20 transformants per microgram of transforming DNA (Punt et al. 1987). Gene manipulation using alteration of genes within the natural genome has allowed this organism to be utilised in food and fibre production and modification. Database resources for *A. niger* build upon molecular resources and include an extensive expressed sequence database (Semova et al. 2006) as well as public systems biology resources (Andersen et al. 2008) based on metabolic integration.

For all *Aspergillus* species, shared data resources have an increasing importance and have grown exponentially. The initial genome analysis was fundamentally comparative, and the Broad online data resources emphasised this comparative aspect, although like other fungal genome resources the Broad *Aspergillus* site is now only available as an online archive (http://archive.broadinstitute.org/ftp/pub/annotation/fungi/aspergillus/). Other shared genome resources for *Aspergillus* include the *Aspergillus* site (http://www.aspergillus.org.uk/) as well as increasing amounts of content at fungiDB (Stajich et al. 2012). The NCBI Short Read Archive includes over 1600 projects using *Aspergillus* including transcriptome and genome sequences of diverse species as well as multiple projects with the reference genome strain FGSC A4 (Pontecorvo et al. 1953b). Because Aspergilli are economically important, there is significant data regarding applied use of these organisms. The US PTO lists over 11,500 patents that include ‘*Aspergillus*’ making this a dense information resource (Table 1). With over 1300 patents citing *Aspergillus*, 2013 is the year with the most patents for Aspergilli.

## Pathogenic Aspergilli

More recently researchers have turned their attention to the human pathogen *Aspergillus fumigatus (*Nierman et al. 2005). While this is not a model system, it is an important research system and the demonstration of sexuality in the lab (O’Gorman et al. 2009) built upon the observation of mating type genes and recombination in a population of wild isolates (Paoletti et al. 2005). With an emphasis on pathogenicity (Fedorova et al. 2008), drug resistance (Mellado et al. 2007; Vermeulen et al. 2013), and population genetics (Araujo et al. 2010), great strides have been made in understanding the biology of this common environmental fungus . Similarly, plant pathogenic *Aspergillus* species including *A. parasiticus* and *A. flavus* have been the subject of intense study (Yu et al. 2008) largely because of their production of toxins in the field and in post-harvest storage (Klich 2007).

### Trichoderma reesei

Originally isolated on cotton canvas tents in the Solomon islands, the first widely utilised strain of *T. reesei* (then, *T. viridae*), QM6a (Reese et al. 1950) was characterised extensively in defining the number and types of cellulase enzymes using biochemical approaches. Because the production of cellulase enzymes was subject to feedback inhibition by liberated sugars (Mandels & Reese 1957), mutagenesis was undertaken to generate strains which produced high levels of cellulose degrading enzymes for industrial applications (Mandels et al. 1971). Originally generating the strain QM9123, this approach was continued to produce the strain RUT-C30 (Montenecourt et al. 1980) which produced 15-fold higher titres of cellulase than the progenitor strains. The three-step mutagenesis protocol used in generating RUT-C30 was ultimately demonstrated to have generated a large genome deletion (Seidl et al. 2008). Another significant development in *Trichoderma* was the early demonstration that it was a suitable platform for the production of active calf chymosin (Harkki et al. 1989).

While primarily envisioned as a tool for producing enzymes for saccharification of cellulose prior to fermentation into ethanol, *Trichoderma* strains have found diverse application as the sources of enzymes for generating fungal protoplasts (Peberdy 1979), clarifying wines (Villettaz 1984), and for other species of *Trichoderma*, as biocontrol agents for the prevention of fungal infection of plants (Papavizas 1985; Fravel 2005). With nearly 3800 entries in the USPTO database (Table 1), *Trichoderma* is an important organism for biotechnology and applied uses including applications of *Trichoderma* that depend largely on the ability to produce and secrete large quantities of enzymes for industry (Druzhinina & Kubicek 2016), as well as for clinical or pharmaceutical applications (Smith et al. 2014) and especially cell-wall degrading enzymes (Peberdy 1979). This last application has had significant impact on the advance of fungal genetics as the use of cell wall degrading enzymes for protoplasting has been foundational for the development of molecular genetic transformation approaches for diverse fungi (Fincham 1989). Similarly, the elucidation of fungal karyotypes using pulsed field gel electrophoresis depended upon the ability to generate high quality protoplasts (Schwartz & Cantor 1984). While this remains true for many fungi, the demonstration that the protoplasting step could be omitted in the preparation of samples for karyotyping (McCluskey et al. 1990) meant that some fungi which were not amenable to protoplasting could be studied by the pulsed field approach (Crouch 1992). The *Trichoderma* karyotype was shown to be comprised of six large chromosomes (Gilly & Sands 1991) similar to karyotypes of *Neurospora* (Orbach et al. 1988) and other filamentous ascomycetes (Mills & McCluskey 1990). The genome sequence for the reference strain of *T. reesei* (Martinez et al. 2008) indicated that biomass degraded enzymes were clustered in the genome and served as the basis for early resequencing studies on high cellulase-producing strains (Le Crom et al. 2009; Koike et al. 2013).

Originally studied as an imperfect fungus, recent tools have allowed genetic analysis (Seidl et al. 2009; Chen et al. 2012; Seibel et al. 2012) allowing strain improvement (Seidl & Seiboth 2010). Similarly, the development of molecular tools for manipulating strains, including gene knockouts, auxotrophic mutants and complementary plasmids, and strains deficient in non-homologous end-joining allowing targeted transformation (Schuster et al. 2012) has meant that *Trichoderma* is as easily manipulated as historical model systems such as *N. crassa* (Dunlap et al. 2007) and *A. nidulans*. To that end, diverse *T. reesei* strains are available from public collections. While the FGSC holds only a few strains from genome sequencing programmes, the straininfo.net resource lists 62 strains of *T. reesei* (among nearly 2,000 *Trichoderma* strains globally). The NRRL collection holds 22 *Trichoderma* strains, of which only three are *T. reesei*. The CBS collection holds 10 *T. reesei* strain among holdings of over 700 Trichoderma strains in total. The Budapest University of Technology and Economics has a culture collection of *Trichoderma* including 1100 *Trichoderma* strains from 33 species (Bissett et al. 2015).

Because other *Trichoderma* species are studied for different applications, diverse data resources for *Trichoderma* research exist. Among them, the secondary metabolite database (https://peptaibiotics-database.boku.ac.at/django/) is unique for fungal model systems. With catalogues of over 1000 metabolites from 20 organisms, this database is useful for studies of mycotoxicity, biocontrol, and for expanded understanding of the genome-to-metabolome relationship. Similarly, to facilitate the understanding of *Trichoderma*, a public taxonomy database at http://www.isth.info/spans the transition from morphological taxonomy to molecular taxonomy and includes barcode sequences and protocols, although it has not been updated recently. Genome data supporting the *Trichoderma* research community include publicly available assembled genome sequences for several species (Martinez et al. 2008; Druzhinina et al. 2011; Kubicek et al. 2011). While not as prolific as for other fungi, public sequence resources at the NCBI Short Read Archive include 76 genome sequence and one transcriptome sequence. The US DOE JGI (http://genome.jgi.doe.gov/) includes nine sequencing projects on seven different species and these data resources will grow as more strains are subject to resequencing.

## Plant pathogenic fungi and mushrooms

With roots as models for mating type genetics, both *Ustilago maydis* (Holliday 1961; Kronstad 2008) and *Schizophyllum commune* (Raper & Miles 1958; Ohm et al. 2010) were characterised extensively in the classical genetics era and the role of these fungi as plant pathogens was not the driving force in their utilisation as model systems. Similarly, mating was studied in the saprophytic basidiomycete mushroom *Coprinopsis cinerea* and this system was ultimately exploited more for its synchronised meiosis than for its other features (Casselton & Kües 2007; Stajich et al. 2010). The rice pathogen, *Magnaporthe grisea*, has been characterised extensively, from the production of a molecular genetic map (Dioh et al. 2000), through comparative genome analysis (Donofrio et al. 2014), and detailed analysis of the cell biology of infection (Giraldo et al. 2013). While studied explicitly to understand its ability to cause disease in rice, *M. grisea* has emerged as a tractable model to understand the biology of the wheat-infecting variety of this fungus which has tremendous potential to impact wheat production in North America, Europe, and Asia (Pieck et al. 2017). Finally, fungi in the genera *Fusarium* produce toxins and cause disease in plants, animals, and even humans, are useful in industrial biotechnology, and are even used directly in producing food for humans (Ma et al. 2013). Fusaria, including *F. graminearum, F. oxysporum, F. verticillioides, F. moniliformis*, and *F. solani* have been studied with a number of techniques including molecular genetic map construction (Jurgenson et al. 2002), electrophoretic karyotyping (Migheli et al. 1993; VanEtten et al. 1998), and ultimately by comparative genomic analysis (Ma et al. 2010; Sperschneider et al. 2015). Because these are more accurately research systems and not model systems, they are not considered further in this contribution.

## Discussion and conclusion

The advance of filamentous fungi for biotechnology has benefitted greatly from the public availability of shared research resources. From wild and mutant strains to gene libraries, plasmids, and targeted gene deletion strains, resource collections like the FGSC, as well as reference collections like the American Type Culture Collection and the Centraalbureau voor Schimmelcultures in the Netherlands, and importantly, the USDA NRRL collection, provide open access to well-characterised research materials for modest fees. As a global system for insuring fair and equitable access to genetic resources under the Nagoya Protocol becomes the dominant paradigm (Dedeurwaerdere et al. 2016), the access to validated materials with sound provenance will increase the ability to implement modern biotechnological applications for pharmaceutical, industrial, and agricultural uses. The Convention on Biological Diversity mandates that every country develop an *ex situ* microbial germplasm repository and the formalisation of these public resources should increase the access to well-characterised microbial resources (Secretariat 1992). Because much genetic research with fungal model systems has evolved into genomics research, shared data resources such as the JGI MycoCOSM system (Grigoriev et al. 2013) and fungiDB (Stajich et al. 2012) are increasingly important. As more fungi, beyond model systems, enter the big data realm, other online resources, such as the JGI Knowledge Base (Palumbo et al. 2014), will become more important for fungal genetics. The amount of data supporting use of these model systems has grown from tens to dozens of unique characters to thousands of full, 30–50 Megabase genomes representing growth of 10–12 orders of magnitude. This proliferation of data will greatly benefit from systems biology approaches facilitated by the use of the integrated knowledge management systems.

The impact of model systems has been undeniable although some systems have been more persistent than others. Clearly the availability of resources through public repositories can assure that resources from one era are available for development of novel applications when technology matures. Much as the auxotrophic mutants of *Ustilago* (Perkins 1949b) were not useful in the modern molecular genetics era (Djamei & Kahmann 2012), some resources developed to study specific questions, for example, mutagenesis (De Serres 1958; De Serres & Webber 1997), chromosome re-arrangements (Perkins 1962a), or intragenic recombination (Suyama & Bonner 1964) are now only utilised in special circumstances. *A. nidulans* has been a model for important applied systems including industrial, agricultural, and medical settings (Goldman & Osmani 2007). With diverse tools and an organised community, *A. nidulans* will continue to impact diverse areas of biology (Todd et al. 2007). Similarly, the impact of *N. crassa* as a model cannot be underappreciated (Davis 2004). With new contributions in understanding photobiology and circadian rhythms (Baker et al. 2012), epigenetics and genome defence (Aramayo & Selker 2013), and following on the demonstration that *N. crassa* has an epiphytic growth phase (Kuo et al. 2014), these important models have more to teach us.

The use of these fungi as industrial organisms including *A. oryzae, A. niger*, and *T. reesei* will continue to have impact beyond their immediate research areas. As there are multiple important species of *Aspergillus* that are utilised in industry and agriculture, and as the increasing impact of *A. fumigatus* in clinical settings is understood, the model system *A. nidulans* will have continuing relevance. Similarly for the diverse species of *Trichoderma* which are used in agriculture and industry, and which have close relatives used in food and pharmaceutical production, the age of model organisms is not over. Diverse research systems can inform their approaches to research directions by learning from these models. Research with plant pathogenic fungi such as *Fusarium, Magnaporthe*, or diverse Dothidiomycetes, and with clinically relevant fungi such as *Cryptococcus*, *Trichoderma*, or *Candida*, can all benefit from the development of diverse data resources supporting model organism research.
